# Viral microRNA inhibition enhances antiviral immunity by modulating corneal inflammatory and resolution pathways in HSV-1 induced keratitis

**DOI:** 10.1016/j.exer.2026.110903

**Published:** 2026-02-04

**Authors:** Chandrashekhar D. Patil, Raza Ali Naqvi, Araceli Valverde, Hemant Borase, Afsar R. Naqvi, Deepak Shukla

**Affiliations:** a Department of Ophthalmology and Visual Sciences, College of Medicine, University of Illinois Chicago, Chicago, IL, 60612, USA; b Department of Periodontics, College of Dentistry, University of Illinois Chicago, Chicago, IL, 60612, USA; c Department of Microbiology and Immunology, College of Medicine, University of Illinois Chicago, Chicago, IL, 60612, USA

**Keywords:** Herpes simplex virus-1, microRNAs, Ocular infection, Immunity, Resolution

## Abstract

Herpes simplex virus type 1 (HSV-1) is a leading cause of infectious corneal blindness worldwide. Viral persistence and disease severity are strongly influenced by the virus's ability to modulate host immune responses; however, the mechanisms by which HSV-1 alters corneal immunity are incompletely understood. In particular, the role of virus-encoded microRNAs (v-miRs) in shaping corneal immune responses during herpes simplex infection remains unclear. We previosuly showed that inhibition of selected HSV-1 v-miRs reduced viral replication and disease severity in a mouse model of ocular infection. Building on these findings, the present study investigated how v-miR inhibition affects corneal immune responses. Using corneal tissue RNA from HSV-1-infected mice, we performed an immune profiling PCR array by analyzing the expression of 88 genes associated with immune cell markers and polarization states. Topical inhibition of miR-H1–5p, miR-H3–3p, and miR-H6–3p resulted in distinct patterns of immune gene expression compared with the control treatment. Inhibition of these v-miRs altered 16, 31, and 57 immune-related genes, respectively, spanning both myeloid- and lymphoidassociated pathways. Notably, the anti-inflammatory genes *Arg1* and *Il10* were consistently upregulated across all v-miR inhibitor-treated groups. In parallel, increased expression of the pro-resolution enzymes *15-lox* and *Alox5* suggested enhanced engagement of resolution pathways. Mechanistic studies demonstrated that v-miRs directly target immune regulatory genes through binding sites within their untranslated regions. Together, these findings suggest that HSV-1 v-miRs contribute to corneal immunopathology by suppressing anti-inflammatory and pro-resolving immune pathways, and that targeted inhibition of v-miRs may promote immune resolution during HSV-1–induced keratitis.

## 1. Introduction

Herpes simplex virus 1 (HSV-1)-infection-induced keratitis (HSK) is a major cause of vision loss worldwide, with an estimated 1.5 million new cases annually (Barron et al., 1994; Farooq and Shukla, 2012; Shah et al., 2019; Wilhelmus et al., 1996). Following primary infection, HSV-1 establishes latency in the trigeminal ganglion and periodically reactivates, seeding the ocular surface and stroma and ultimately causing cumulative, often irreversible, structural damage to the cornea (Al-Dujaili et al., 2011; Farooq and Shukla, 2011; Held and Derfuss, 2011). Clinically, topical corticosteroids primarily suppress inflammation and alleviate symptoms but do not eliminate viral replication (Arain et al., 2015; Mancini et al., 2025), and acyclovir-resistant HSV-1 strains are increasingly reported, underscoring the limitations of current antiviral strategies (Muluneh et al., 2013; Schalkwijk et al., 2022). Together, these observations emphasize the need for alternative approaches that both inhibit viral replication and promote immune resolution to prevent progressive corneal injury.

HSV-1 typically persists as a lifelong, recurrent, often subclinical infection, implying that the virus must continuously evade or subvert host immune surveillance (Su et al., 2016; Sutter et al., 2025). HSV-1 encodes more than 80 protein-coding genes (Kato et al., 2020; Whisnant et al., 2020) and at least 25 mature microRNAs (miRNAs) (Griffiths-Jones et al., 2008). miRNAs are small (~19–24 nt) noncoding RNAs that bind complementary sequences in target transcripts, leading to mRNA degradation or translational repression. Virus-encoded miRNAs (v-miRs) are critical determinants of pathogenesis because they can simultaneously regulate both viral and host gene networks (Cui et al., 2006; Jurak et al., 2010; Munson and Burch, 2012; Naqvi et al., 2018; Naqvi et al., 2025; Sharma et al., 2025; Umbach et al., 2009). Numerous HSV-1 proteins have been shown to modulate host immunity by targeting key innate and adaptive signaling pathways; for example, UL42 and ICP0 interact with p65/RelA and p50/NF-κB1, blocking NF-κB nuclear translocation and thereby reducing antiviral cytokine production) (Zhang et al. 2013a, 2013b). Beyond these classical immune evasion mechanisms, viruses also interfere with the active resolution of inflammation by disrupting the lipid mediator “class switch” from pro-inflammatory eicosanoids to specialized pro-resolving mediators (SPMs), thereby prolonging neutrophil recruitment and delaying tissue repair (Irún et al., 2023; Palmas et al., 2021). By downregulating pro-resolving enzymes such as 15-lipoxygenase (15-LOX) and 5-lipoxygenase (ALOX5/5-LO), or attenuating their upstream signals, viruses diminish the biosynthesis of resolvins, protectins, maresins, and lipoxins (Gosselin and Borgeat, 1997; Norling et al., 2017) that normally terminate inflammation and enhance efferocytosis (Ke et al., 2025; Salina et al., 2022). Viral proteins and miRNAs can further reprogram macrophages, dendritic cells, and T cells into pro-inflammatory, chronically activated, and dysfunctional states. For example, the Epstein-Barr virus latent membrane protein 1 (LMP1) is a CD40 mimic that constitutively activates NF-κB, JNK, and p38 in B cells, driving sustained production of pro-inflammatory cytokines (Eliopoulos et al., 1999; Gires et al., 1997) and anti-apoptotic signaling (Izumi et al., 1999), thereby impairing resolution dynamics. While the functions of many viral protein-coding genes are well characterized, the contribution of HSV-1–encoded miRNAs to ocular pathogenesis, immune modulation, and failure of resolution remains poorly defined.

Virus-encoded miRNAs are critical determinants of pathogenesis because they can simultaneously regulate viral and host gene networks (Ghosh et al., 2009; Grundhoff and Sullivan, 2011). Apparently, only a subset of HSV-1 encoded miRNAs has been functionally linked to host–pathogen interactions. For instance, HSV-1 miR-H2–3p targets the DNA sensor DDX41, facilitating viral replication while reducing IFN-β and Mx1 production (Duan et al., 2019). Additionally, miR-H8 down-regulates cell-surface MHC class I expression by targeting the GPI-anchoring pathway, thereby diminishing antigen presentation (Enk et al., 2016). Oncogenic Kaposi's sarcoma–associated herpesvirus (KSHV) encoded miR-K12–11, a functional ortholog of host miR-155, drives a pro-inflammatory, proliferative program in B cells at the expense of regulatory and pro-resolving pathways (Boss et al., 2011). However, while acute inflammation during viral infection can eliminate infected cells and then subside, failure to mount an effective pro-resolution response can prevent proper shutdown of inflammation, promote viral persistence, and in severe cases contribute to poor clinical outcomes (Ferri et al., 2023; Tavares et al., 2023). Resolution of inflammation is orchestrated by SPMs and their biosynthetic enzymes and receptors. Prior studies in other inflammatory settings have shown that enzymes such as 15-LOX and ALOX5/5-LO contribute to the generation of lipid mediators that shape leukocyte trafficking, efferocytosis, and the restoration of tissue homeostasis (Basil and Levy, 2016; Serhan et al., 2011). In parallel, pro-resolving signaling can be mediated through receptors implicated in lipid mediator responses, including BLT1 and ALX/FPR2, which participate in immune regulation across multiple tissues (Bannenberg and Serhan, 2010). The role of HSV-1 miRNA-mediated disruption of corneal immune homeostasis and resolution mediators remains largely understudied.

We previously demonstrated that *in vivo* inhibition of multiple HSV-1 v-miRs significantly suppresses viral replication and persistence (Patil et al., 2025). Building on this, the present study investigates how candidate v-miR inhibition affects the immunopathology of HSV-1–induced keratitis, with a focus on pathways related to inflammation and resolution. Using a targeted PCR array, we performed comprehensive immune profiling of the myeloid and lymphoid compartments in HSV-1–infected corneas treated with v-miR inhibitors and identified novel host gene networks regulated by these miRNAs that are associated with anti-inflammatory and pro-resolving responses. Our findings demonstrate that *in vivo* v-miR inhibition not only restricts viral replication but also promotes the emergence of anti-inflammatory immune subsets and resolution-linked gene signatures, thereby supporting the restoration of corneal immune homeostasis and improved disease outcomes.

## 2. Materials and methods

### 2.1. Reagents

The HSV-1 (McKrae) strain used in this study to infect the mouse eye was provided by Prof. Homayon Ghiasi's laboratory at Cedars Sinai, Los Angeles, CA. HSV-1 (McKrae) was propagated, titered on Vero cells, and stored at −80 °C. Viral miRNA inhibition was performed using custom-designed locked nucleic acid (LNA) antisense oligonucleotides (miR-CURY LNA miRNA Custom Inhibitors; QIAGEN). Mature viral miRNA sequences were obtained from validated annotations and submitted to QIAGEN for custom design. Inhibitors were designed to exhibit perfect complementarity to the mature miRNA, thereby sequestering target miRNAs and blocking downstream activity. Oligonucleotides were supplied HPLC-purified, reconstituted in nuclease-free water, aliquoted, and stored at −15 to −30 °C until use.

### 2.2. Mouse ocular HSV-1 infection model, v-miR inhibitor treatment, and corneal RNA preparation

The mouse ocular HSV-1 infection model and topical delivery of viral microRNA (v-miR) inhibitors used in this study were previously described in our earlier publication (Patil et al., 2025). No new animal infections or treatments were conducted for the present study. Instead, RNA extracted from archived corneal tissue from the original experimental cohort was used for focused downstream analyses of immune gene expression. Briefly, male C57BL/6 mice (6–8 weeks of age) were anesthetized with ketamine (100 mg/kg) and xylazine (5 mg/kg), and the right eye was topically anesthetized with proparacaine hydrochloride. Corneal epithelial debridement was performed using a sterile 30-gauge needle, followed by ocular infection with HSV-1 strain McKrae at a dose of 1 × 10^5 plaque-forming units (PFU) per eye in a total volume of 5 μL.

To evaluate the impact of v-miR inhibition on HSV-1-induced corneal immunopathology, HSV-1-specific miRNA inhibitors targeting miR-H1–5p, miR-H2–5p, miR-H3–3p, miRH4–3p, miR-H5–3p, miR-H6–3p, miR-H27, miR-H7–3p, and miR-H17 or a non-targeting control inhibitor were complexed with Lipofectamine 2000, diluted in 1 × phosphate-buffered saline (PBS), and administered topically to infected eyes beginning at 3 h postinfection. Treatments were applied three times daily through 7 days postinfection. While multiple v-miR inhibitors were evaluated in the original study, the present analysis focused on corneal transcript data from mice treated with miR-H1–5p, miR-H3–3p, or miR-H6–3p inhibitors, based on their previously observed disease-modifying effects. To determine the effective corneal delivery of the LNA inhibitors, we performed *in vivo* uptake experiments using a Cy3-labeled non-targeting control inhibitor (Supplementary Fig. 1).

For immune gene expression analysis, mice were euthanized at 8 days postinfection (dpi), and whole eyes were collected. Corneas were dissected under a stereomicroscope using sterile instruments, sonicated (with 5-s pulses at 20% amplitude for a total of 30 s), and stored in 700 μL TRIzol reagent and total RNA was isolated using the miRNeasy Micro Kit. RNA samples were stored at −80 °C until further processing. Samples were maintained under RNase-free conditions and were not subjected to repeated freeze-thaw cycles. In the present study, these archived RNA samples were used to focus downstream immune gene expression analyses. Prior to analysis, RNA quality was re-evaluated to ensure established integrity and purity criteria. We used 250 ng of RNA, which was reverse-transcribed into cDNA using the High-Capacity cDNA Reverse Transcription Kit according to the manufacturer's instructions. RT-qPCR was performed on a StepOne 7500 (Applied Biosystems, USA) using SYBR Green Master Mix (Applied Biosystems, USA) to assess *15-lox*, *Alox5*, *blt1* and *alx/fpr2* genes. *Gapdh* was used as a housekeeping gene. The primer sequences are listed in Supplementary Table 1. The Ct values of three replicates were analyzed to calculate the fold change using the 2 ^−ΔΔCt^ method.

After that, a targeted PCR array was performed (containing 88 primer sets directed against immune cell surface and polarization markers and 8 housekeeping primer sets) (Real Time Primers, LLC, Elkins Park, PA) on a StepOne 7500 thermocycler (Applied Biosystems, Carlsbad, CA). The data were analyzed using the QiagenGeneGlobe Data Analysis Center (Source:https://www.qiagen.com/us/shop/genesand-pathways/data-analysis-center-overview-page/). CT values of all genes were normalized to those of GAPDH (a housekeeping control) prior to calculating the final fold change values.

### 2.3. Primary murine bone-marrow monocyte isolation and macrophage culture

The bone marrow from femurs and tibias of 8–12-week-old mice was flushed using a 26-gauge needle preloaded with complete DMEM (DMEM supplemented with 10% FBS and 1% penicillin–streptomycin) and collected into 15 mL conical tubes. Cells were centrifuged at 800×*g* for 5 min at room temperature. The supernatant was discarded, and the pellet was resuspended in 1 mL of MACS buffer. To remove red blood cell contamination, 9 mL ACK lysis buffer was added, and samples were incubated briefly, followed by centrifugation at 800×*g* for 5 min. The supernatant was discarded, and ACK lysis was repeated twice until the pellet showed no visible red blood cell contamination. For macrophage differentiation, bone marrow cells were cultured following our previously published protocol. Briefly, cells were maintained in complete medium supplemented with recombinant murine GM-CSF (rmuGM-CSF; 1000 U/mL; PeproTech), and differentiation was assessed by flow cytometry on day 7. Macrophage markers CD11b and F4/80 (CD11b^+^F4/80^+^) indicate robust macrophage differentiation (>95%).

### 2.4. Flow cytometry

To ensure macrophage differentiation, the cells were removed from the 24-well plate using an enzyme-free cell dissociation buffer (Ther-moFisher Scientific). Single cell suspension was gently washed with 1xPBS (1% BSA w/v) two times and stained with Pacific Blue^™^ anti-mouse/human CD11b Antibody (Clone: M1/70; BioLegend) and PE anti-mouse F4/80 Antibody (Clone: W20065B; BioLegend) for 30 min at 4 °C. After that, the cells were washed twice with 1x PBS (1% BSA w/v), fixed in 2% paraformaldehyde, and then resuspended in 180 μL for data acquisition in the flow cytometer (Cytoflex).

For the detection of phosphorylated proteins, CD11b^+^F4/80^+^ macrophages were transfected with viral miRNA mimics or a control mimic (final concentration, 25 nM). After 24 h, cells were stimulated with a TLR9 agonist, and 24 h later, they were harvested to quantify the phosphorylation of TLR9 downstream transcription factors by intracellular phospho-flow cytometry, as previously described by our group (Naqvi et al., 2025).

Harvested CD11b^+^F4/80^+^ cells were washed twice with RPMI +1% BSA, fixed in Cytofix buffer (BD Biosciences) for 30 min at 4 °C, washed twice, and permeabilized using Perm/Wash buffer I for 20 min at room temperature. Cells were then washed and stained with antibodies against phosphorylated signaling proteins: phospho-NF-κB p65 (BV421-conjugated anti-NF-κB [p65], clone K10–895.12.50, BD Biosciences) and phospho-IRF7 (Alexa Fluor 647 647-conjugated anti-IRF-7 [pS477/pS479], clone K47–671, BD Biosciences) for 45 min at 4 °C. After staining, cells were washed three times with RPMI +1% BSA (each wash at 600×*g* for 10 min) and resuspended in 180 μL RPMI + 1% BSA for acquisition on a CytoFLEX flow cytometer. Data were analyzed using FlowJo v10.8.1 (Tree Star, Ashland, OR). FlowJo_v10.8.1 software (Tree Star, Ashland, OR) was used to analyze the flow cytometry data.

### 2.5. MiRNA target prediction of differentially upregulated genes

To identify miR-H1–5p, miR-H3–3p and miR-H6–3p gene targets with high confidence, we selected upregulated genes in animals treated with the miRNA inhibitors. The 3 ′ UTRs of these genes were extracted using the BioMart tool on Ensembl (http://www.ensembl.org/biomart/martview/aa867419c3c6fd64f94af6d4a6549d3c) as previously described.^45^ miRNA target 3′UTR interaction was assessed by the target prediction tool RNA Hybrid software (https://bibiserv2.cebitec.uni-bielefeld.de/rnahybrid?id¼rnahybrid_view_submission). The procured 3 ′ UTR sequences and miR-H6–3p and miR-H27 sequences (extracted from miRBase v.21) were provided as input for RNA Hybrid analysis. The stringency parameters were set up for individual sequences, and we opted for three hits per target to highlight any probable miRNA binding sequence present on the target. We considered the following parameters to select putative miR-regulated genes: (i) There should be high sequence complementarity in the seed region (positions 2–8 nt from 5′ of miRNA), with only 1 mismatch allowed (ii). For stringency, we picked miR-target interactions where more than 11 nts of the miR sequence are involved in the interaction. (iii) If there is any mismatch in the seed regions, this should be compensated for by strong binding beyond the seed region. (iv) The bulge in the interaction region should not involve more than 3 nucleotides. (v) Entropy of the miR-target interaction was set at a stringent level with a cut-off of <22 kcal/mol.

### 2.6. Cloning 3′UTR of gene targets

The cloning of target 3 ′ UTRs was performed as previously described (Naqvi et al., 2015). Briefly, the 3′UTRs of IL-10 and Arg1 were PCR-amplified using gene-specific forward and reverse primers and genomic DNA isolated from freshly prepared PBMCs as the template. All primers included engineered *Xho*I and *Not*I restriction sites (Supplementary Table 2). PCR was performed using Phusion DNA polymerase (New England Biolabs, Ipswich, MA, USA). The resulting amplicons were digested with *Xho*I and *Not*I and ligated downstream of the luciferase reporter in the psiCHECK^™^-2 vector (Promega).

### 2.7. Dual luciferase assays

Dual luciferase reporter assays were performed using the Promega Dual Luciferase Kit (Catalog #E1980, Promega, Madison, WI) as previously described (Naqvi et al., 2014, Naqvi et al., 2015; Naqvi et al., 2016). Briefly, HEK293 cells were seeded in 96-well plates at a density of 3×10^4^ cells/well in DMEM supplemented with 10% fetal bovine serum and 1% Pen-Strep. All transfections were performed using 0.5 μL of Lipofectamine (2000) (Invitrogen), 120 ng of dual luciferase reporter control plasmid psiCHECK2 or plasmids containing target genes, and co-transfected with a final concentration of 10 nM and 25 nM of synthetic miR-H1–5p, miR-H3–3p, and miR-H6–3p or control mimics (Qiagen). After 36 h, the cells were lysed in passive lysis buffer (Promega Corporation), and dual luciferase assays were performed using a mul-tilabel reader (Victor X5, PerkinElmer Health Sciences Inc., Shelton, CT, USA). For each reporter 3′UTR construct, the Rluc/Fluc value obtained was normalized to the value obtained for the empty vector (EV) co-transfected with the same miRNA mimic. The values obtained were plotted as histograms, with EV set at 1.

### 2.8. Statistical analysis

The statistical analyses were performed using GraphPad Prism software (version 4.0). Significance was defined based on exceeding different p-value thresholds: * = p < 0.05, ** = p < 0.01, *** = p <0.001, **** = p < 0.0001.

## 3. Results

### 3.1. Mice treated with inhibitors of HSV-1 encoded miRNAs exhibit distinct immune infiltration profiles

Viral miRNA reshapes the host immune landscape to avoid clearance by innate and adaptive immune responses (Castello et al., 2024; Skalsky and Cullen, 2010; Speck and Ganem, 2010). The *in* vivo role of HSV-1 miRs in immune suppression in the cornea, however, remains to be explored. We previously showed that topical inhibition of selected v-miRs attenuates HSV-1 viral replication, disease burden and immune activity in lymph nodes in a murine model of ocular keratitis (Patil et al., 2025). In this study, we investigated whether these HSV-1 miRNA inhibitors influence the immune environment of the cornea. To address this, we processed RNA from the corneas of HSV-1–infected mice treated topically with the three most potent inhibitors targeting miR-H1–5p, miR-H3–3p, and miR-H6–3p to determine whether their therapeutic effect extends beyond antiviral activity to include modulation of local immune responses. The expression profile of 88 genes, including surface markers, cytokines, and transcription factors (TF), was examined using a PCR array to gain insight into the infiltration of immune cells and polarization in the HSV-1-infected cornea. Our results show that each v-miR inhibitor induced a distinct transcriptional program.

**Fig. 1.**
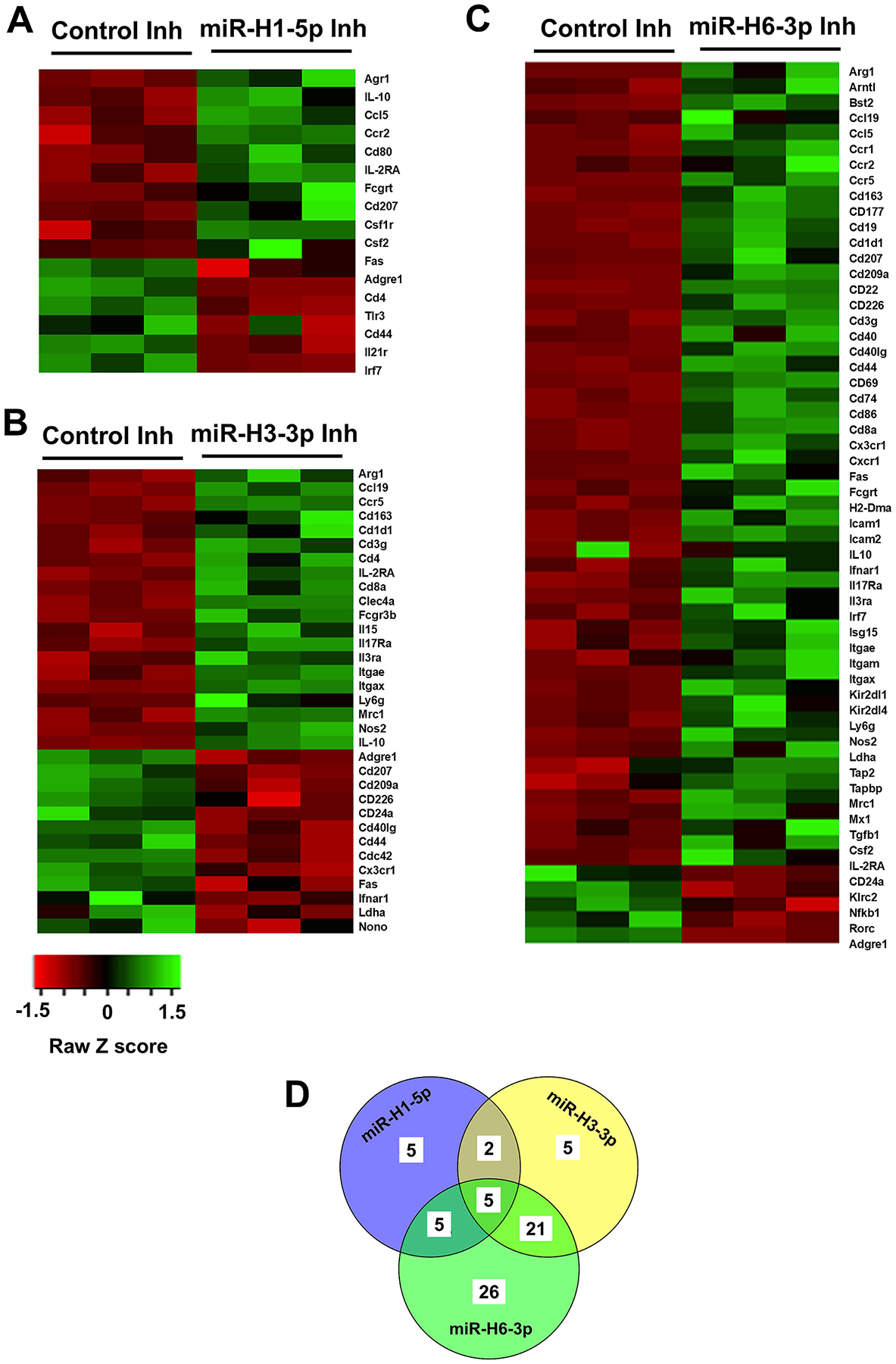
Viral miRNA inhibition reshapes innate and adaptive immune gene signatures in HSV-1–infected murine corneas. Mice were infected with HSV-1 (1 × 10^5^ PFU) following corneal epithelial debridement with a 30-gauge needle. For inhibitor delivery, each viral miRNA inhibitor was complexed with Lipofectamine 2000 and topically administered to the right eye three times daily (10 μM). At 8 days post-infection (dpi), total RNA was isolated from corneas, reverse-transcribed into cDNA, and analyzed using a custom RT-qPCR array profiling 88 immune-related genes. Heatmaps depict the differential expression of transcripts associated with innate and adaptive immune cells and effector molecules in HSV-1-infected corneas treated with (A) a control mimic versus the miR-H1–5p inhibitor (19 genes), (B) a control mimic versus the miR-H3–5p inhibitor (33 genes), and (C) a control mimic versus the miR-H6–3p inhibitor (57 genes). (D) A Venn diagram summarizes the shared and unique genes regulated across the three inhibitor treatments. Fold changes were calculated using GAPDH as the housekeeping control. n = 3 mice per condition.

Inhibition of miR-H1–5p increased expression of chemokine signaling and antigen presentation/co-stimulation genes. Specifically, miR-H1–5p inhibition upregulated six genes *Ccl5*, *Ccr2*, *Cd207*, *Cd80*, *Csf1r*, *Fas*, and *Fcgrt*, and downregulated five genes including *Adgre1*, *Cd4*, *Tlr3*, *Cd44*, and *Il21r* (Fig. 1A; Supplementary Table 1). This suggests that inhibiting miR-H1–5p modulates immune cell recruitment and activation signals while selectively suppressing the expression of leukocyte markers.

In contrast, inhibition of miR-H3–3p elicited a broader immune remodeling, with the simultaneous induction of both myeloid and lymphoid transcriptional signatures. Corneas from miR-H3–3p inhibitor-treated mice showed robust upregulation of 15 genes (p = 0.05), including Arg*1*, *Ccl19*, *Ccr5*, *Cd163*, *Cd1d1*, *Cd3g*, *Cd4*, *Cd8a*, *Clec4a*, *Fcgr3b*, *Il15*, *Il17ra*, *Il3ra*, *Itgae*, *Itgax*, *Ly6g*, *Mrc1*, and *Nos2* (Fig. 1B; Supplementary Table 2). We also observed downregulation of 13 genes, including *Adgre1*, *Cd207*, *Cd209a*, *Cd226*, *Cd24a*, *Cd4*0lg, *Cd44*, *Cdc42*, *Cx3cr1*, *Fas*, *Ifnar1*, *Ldha*, and *Nono*, suggesting that miR-H3–3p inhibition triggers immune activation while suppressing specific trafficking and interferon-associated genes.

Inhibition of miR-H6–3p resulted in the most extensive transcriptional shift, affecting chemokine/trafficking signals and enhancing antigen presentation and immunoregulatory gene profiles. miR-H6–3p inhibition upregulated Arg*1*, *Arntl*, *Bst2*, *Ccl19*, *Ccl5*, *Ccr1*, *Ccr2*, *Ccr5*, *Cd163*, *Cd177*, *Cd19*, *Cd1d1*, *Cd207*, *Cd209a*, *Cd22*, *Cd226*, *Cd3g*, *Cd40*, *Cd4*0lg, *Cd44*, *Cd69*, *Cd74*, *Cd86*, *Cd8a*, *Cx3cr1*, *Cxcr1*, *Fas*, *Fcgrt*, *H2-Dma*, *Icam1*, *Icam2*, *Il10*, *Ifnar1*, and *Il17ra*, while downregulating *Cd24a*, *Klrc2*, *Nfkb1*, *Rorc*, and *Adgre1* (Fig. 1C; Supplementary Table 3). Together, these transcriptional changes suggest coordinated recruitment and activation of immune populations, accompanied by the simultaneous suppression of select lymphoid/NK-associated and inflammatory transcriptional regulators.

The Venn diagram further showed that viral microRNA inhibition impacted expression of both common and unique genes (Fig. 1D). Several genes were consistently affected, including Arg*1*, *Il10*, and *Nos2* (all upregulated) and *Adgre1* (all downregulated) by all three inhibitors, suggesting a shared axis of regulation across these viral microRNAs. Overall, these results demonstrate that inhibition of v-miRs modulates corneal immunity by promoting the expression of anti-inflammatory genes.

### 3.2. Viral miRNA inhibition suppresses immune activation by downregulating NFκB and IRF signaling

Our recent work has shown that ocular HSV-1 infection reshapes the corneal miRNA landscape and is associated with altered TLR9 expression and downstream signaling (Sharma et al., 2025). Because endosomal TLRs (TLR3/7/8/9) sense viral nucleic acids and can be engaged by specific miRNA species in other inflammatory settings, we hypothesized that select HSV-1 v-miRNAs may directly tune TLR-driven signaling in myeloid cells. To examine this, we transfected bone marrow–derived mouse macrophages with HSV-1 miRNA mimics or matched inhibitors and quantified downstream transcription factor activation by phospho-flow.

**Fig. 2.**
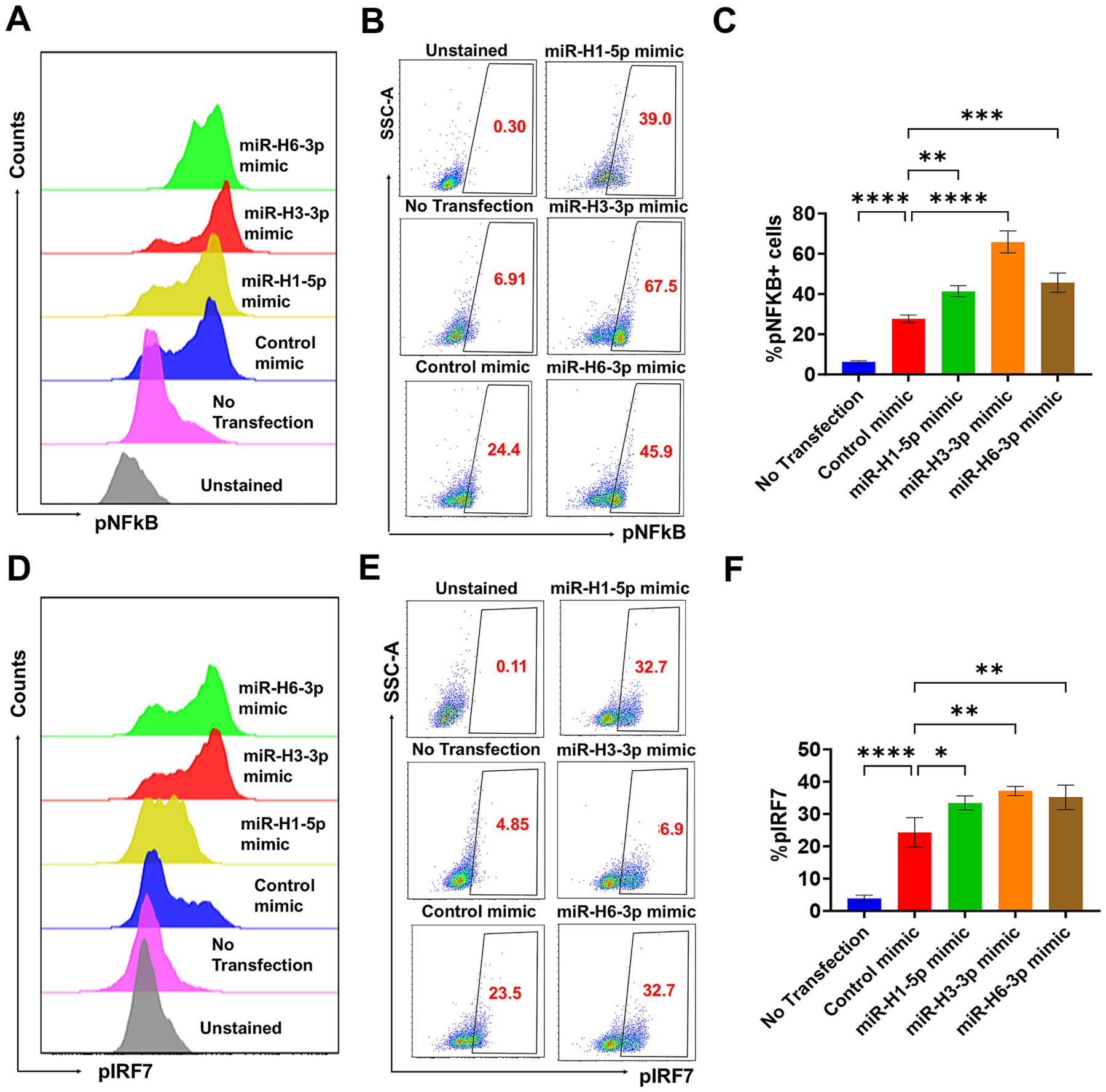
HSV-1–encoded miRNAs activate TLR9-associated downstream signaling in murine macrophages. Bone marrow–derived macrophages were flow-sorted and transfected with commercially available HSV-1 miRNA mimics (25 nM) for miR-H1–5p, miR-H3–3p, and miR-H6–3p, or with the corresponding control mimic. Where indicated, cells were transfected with the matched miRNA inhibitors and compared with their corresponding mimic conditions. After 36 h, cells were challenged with TLR9 agonist ODN2006 (5 μM) for 4 h. Phosphorylation of NF-κB p65 (A-C) and IRF7 (D-F) was assessed by phospho-flow cytometry. Representative scatter plots (A, B, D, E) show phospho-positive populations, with red values indicating the percentage of phospho–NF-κB + or phospho–IRF7+ macrophages. Summary bar graphs report the geometric mean fluorescence intensity (Geo.MFI) for phospho–NF–κB (C) and phospho–IRF7 (F), as quantified in FlowJo v10.9.0. Data are presented as mean ± SD from n = 5 mice per condition. Statistical significance was determined by one-way ANOVA, with p values indicated as p < 0.05, p < 0.01, and p < 0.001.

Macrophages were transfected with mimics or inhibitors of miR-H1–5p, miR-H3–3p, and miR-H6–3p, and phosphorylation of NF-κB and IRF7 was assessed 48 h later using an established phospho-flow approach (Naqvi et al., 2025). Relative to control mimic (31.03 ± 7.1% phospho-NF-κB + cells), transfection with each of the miRNA mimics markedly increased the proportion of phospho–NF-κB + cells: miR-H1–5p: 41.36 ± 3.0%; miR-H3–3p: 65.9 ± 5.4%; and miR-H6–3p: 45.6 ± 4.8% (Fig. 2A–C).

IRF7 phosphorylation also exhibited a similar pattern. Compared with control mimic (23.0 ± 4.5%), miR-H1–5p (33.44 ± 2.1%), miR-H3–3p (37.1 ± 1.4%), and miR-H6–3p (35.13 ± 3.8%) mimic transfected cells showed increased proportion of phosphor-IRF7+ macrophages. Consistent with the NF-κB data, miR-H6–3p had the most pronounced impact on IRF7 phosphorylation (Fig. 2D–F). Together, these results suggest that HSV-1–encoded miRNAs, particularly miR-H1–5p, miR-H3–3p and miR-H6–3p, promote activation of innate signaling pathways plausibly by perturbing TLR9/7-associated signaling, and exogenous miRNA inhibitors can suppress dysregulation by v-miRs.

### 3.3. Viral miRNA inhibition induces pro-resolution markers in HSV-1-infected cornea

In our recent study, topical delivery of HSV-1 v-miRNA inhibitors reduced periocular inflammation in mice infected with HSV-1 (Patil et al., 2025). Considering this, we inquired whether inhibiting specific HSV-1 viral miRNAs actively induces a pro-resolution program in the cornea during infection. This question is clinically relevant due to the limited knowledge of the resolution pathways that can limit HSV-1–driven ocular immunopathology.

**Fig. 3.**
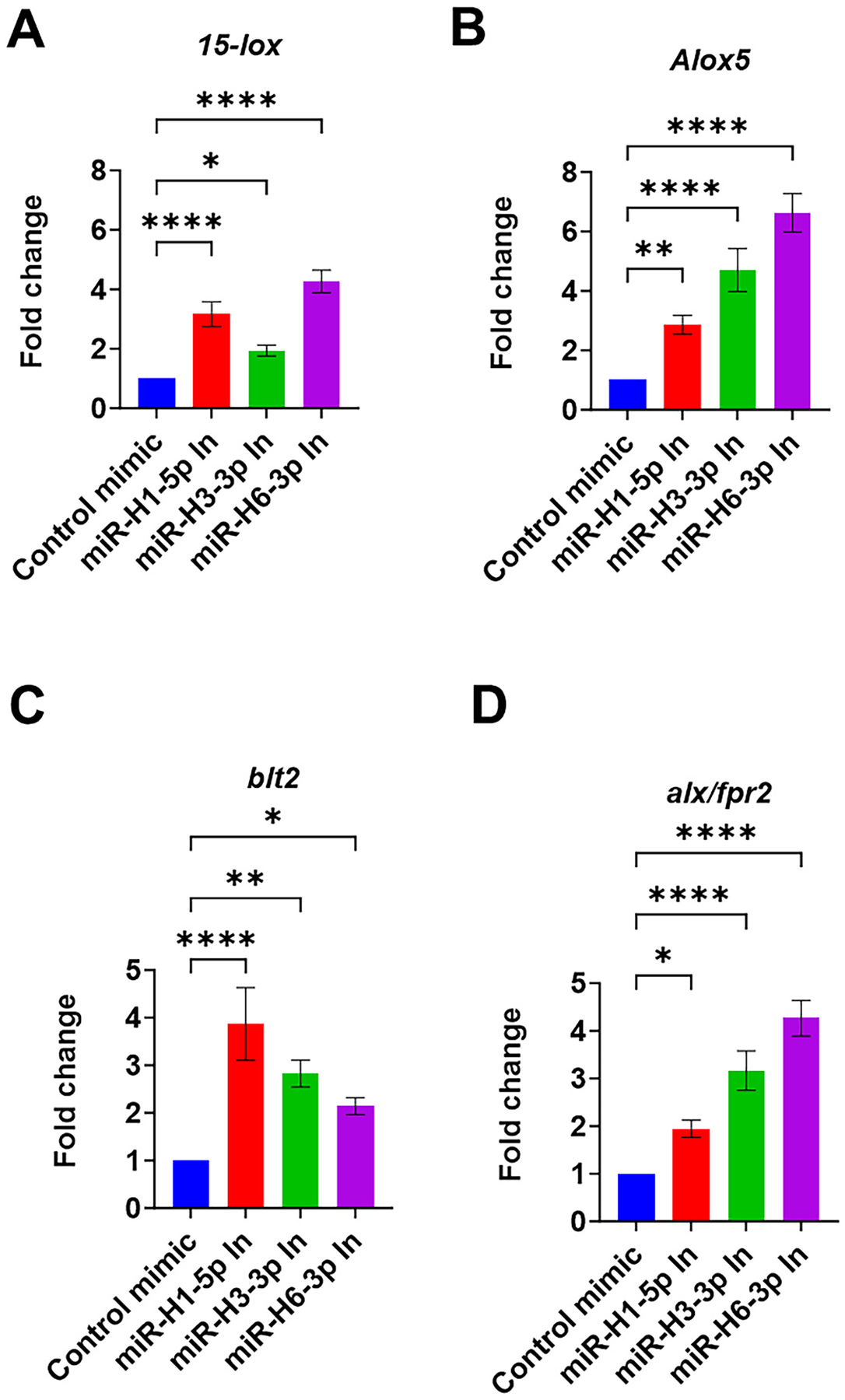
Delivery of HSV-1 v-miRNA inhibitors upregulates resolution-associated pathways in HSV-1–infected corneas. Corneas from HSV-1–infected mice treated with inhibitors targeting HSV-1–encoded miRNAs (miR-H1–5p, miR-H3–3p, and miR-H6–3p) were collected at 8 days post-infection (dpi). Transcript levels of (A) *15-lox*, (B) *alox5*, (C) *blt2*, and (D) *alx/fpr2* were quantified by RT-qPCR and expressed as fold change relative to the control inhibitor group (normalized to the indicated housekeeping gene). Bar graphs were generated in GraphPad Prism 10; each bar represents mean ± SD. Statistical significance was assessed using Student's t-test, with p values denoted as p < 0.05 (*), p < 0.01 (**), and p < 0.001 (***). n = 4 mice per group.

We quantified the gene expression of key resolution-associated enzymes (*15-lox and Alox-5*) and receptors (*blt1* and *alx/fpr2*) in HSV-1-infected corneas following treatment with v-miRNA inhibitors at day 8 post-infection. On day 8, corneas treated with miR-H1–5p, miR-H3–3p, and miR-H6–3p inhibitors showed a selective upregulation of pro-resolution enzymes. Specifically, *15-lox* expression increased by about 3-fold, 2-fold, and 4-fold in corneas treated with miR-H1–5p, miR-H3–3p, and miR-H6–3p inhibitors, respectively, compared with the control inhibitor (Fig. 3A). *Alox5* (5-LO) followed a similar trend, increasing to approximately 3-fold, 5-fold, and 7-fold in corneas treated with miR-H1–5p, miR-H3–3p, and miR-H6–3p inhibitors, respectively, relative to controls (Fig. 3B). Taken together, these results suggest that blocking v-miRNAs pushes the cornea toward a gene-expression profile that aligns with activation of lipid mediator pathways during the later stage of infection.

We next examined receptors involved in lipid mediator signaling. By day 8, *blt1* and *alx/fpr2* expression was higher than in the control mimic in corneas treated with the miR-H1–5p inhibitor (3.86 ± 0.76-fold and 1.94 ± 0.17-fold), the miR-H3–3p inhibitor (2.82 ± 0.29-fold and 3.16 ± 0.41-fold), and the miR-H6–3p inhibitor (2.14 ± 0.35-fold and 4.23 ± 0.49-fold), respectively (Fig. 3C and D). Overall, these data support the notion that HSV-1 v-miR inhibitors promote a pro-resolution transcriptional program in infected corneas, characterized by increased expression of enzymes involved in lipid mediator biosynthesis and their associated receptors. This provides a mechanistic explanation for the previously observed anti-inflammatory phenotype and suggests that v-miR inhibition promotes the resolution of inflammation.

### 3.4. HSV-1 miRNAs directly target anti-inflammatory genes

**Fig. 4.**
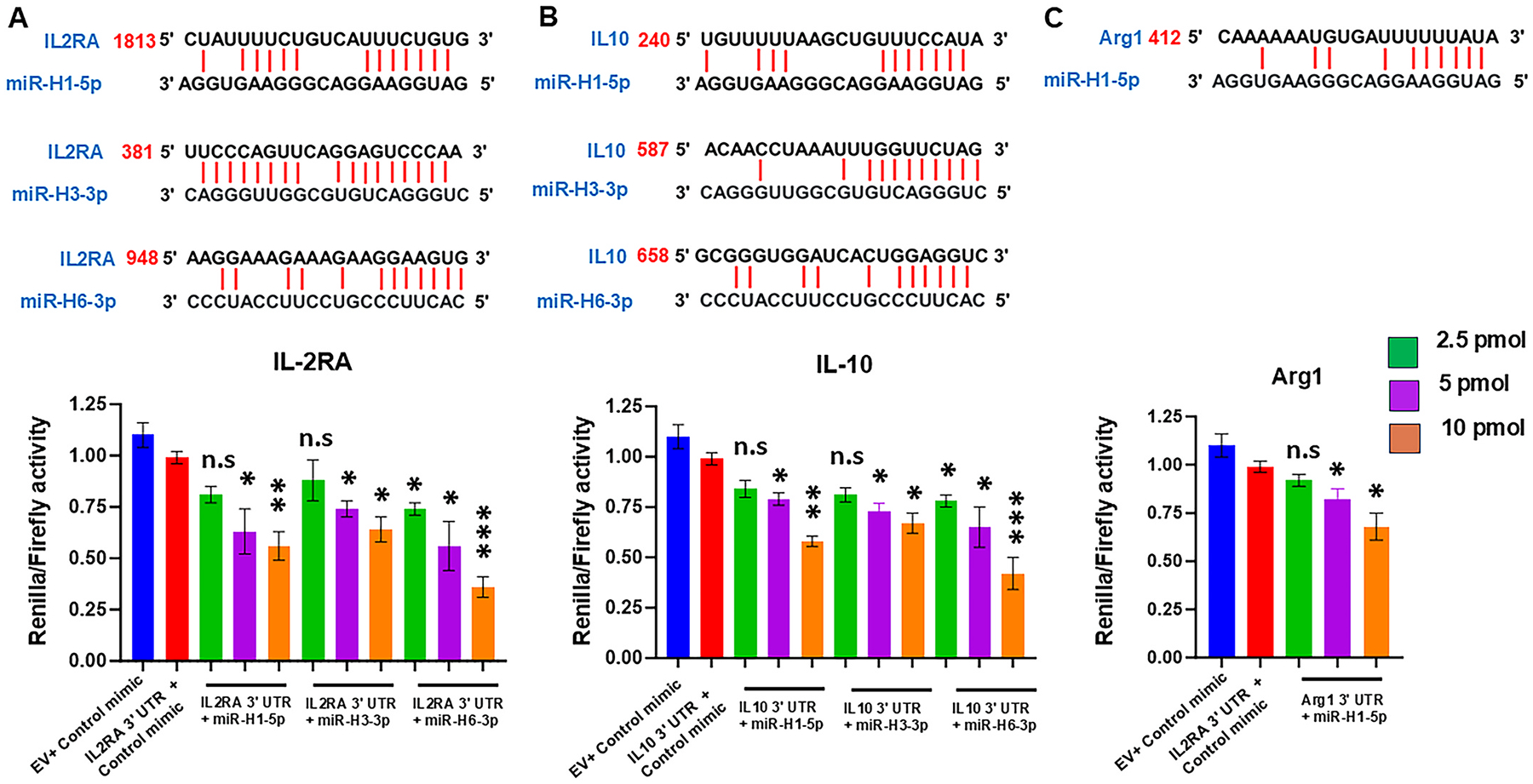
HSV-1 encoded vmiRs directly regulate anti-inflammatory genes. Renilla activity was suppressed via the 3′ UTR of (A-B) Arg1 and (C-D) IL-10 by vmiR mimics, miR-H3–3p and miR-H6–3p, reflecting the putative binding sites of these vmiRs. psiCHECK^™^-2 harboring the 3′UTRs of either Arg1 and IL-10 was co-transfected with vmiR mimics in HEK293 cells. Renilla activity was normalized to firefly activity and the ratios subsequently normalized to empty vector transfected with viral miRNA mimic set as 1. (A–D). Each bar represents the mean ± SEM from three biological replicates. p-values were calculated using One-way ANOVA (**p < 0.01; ***p < 0.0001).

To determine whether HSV-1–encoded v-miRNAs directly regulate anti-inflammatory host genes, we focused on *Arg-1*, *Il-10*, and *Il-*2RA are key immunoregulatory markers implicated in the resolution of inflammation. *Il10* and *Il-*2RA each contain predicted seed-matching sites for all three viral miRNAs, whereas Arg*1* contains a predicted seed-matching site only for miR-H1–5p (Fig. 4A–C; upper panel). In addition, each of the three v-miRs is predicted to bind at a non-overlapping site within the target 3′UTR, suggesting robust targeting of host genes.

To validate these predicted interactions, we employed dual-luciferase reporter assays in HEK293 cells. Cells were co-transfected with psiCHECK^™^-2 constructs containing the human IL2RA, IL-10, or Arg*1* 3′UTR downstream of Renilla luciferase together with HSV-1 v-miRNA mimics (miR-H1–5p, miR-H3–3p, or miR-H6–3p) or the control mimic. Renilla luciferase activity was normalized to firefly luciferase, and values were further normalized to the appropriate control condition (set to 1). We also observed a dose-dependent reduction in Renilla luciferase activity with miR-H1–5p, miR-H3–3p and miR-H6–3p mimics. Our results show that miR-H1–5p, miR-H3–3p and miR-H6–3p mimics significantly suppressed Renilla luciferase activity at higher dose (10 pmol) from the IL-2RA (Fold change: 0.56 ± 0.11, 0.64 ± 0.06, 0.36 ± 0.09), IL-10 (Fold change: 0.58 ± 0.025, 0.67 ± 0.05, 0.42 ± 0.08) 3′UTR reporter, consistent with direct post-transcriptional repression mediated through the predicted target region. Compared with miR-H1–5p and miR-H3–3p, miR-H6–3p exhibited a significantly stronger suppression at the higher mimic dose, indicating greater potency of miR-H6–3p–mediated repression under these conditions. miR-H1–5p mimics significantly reduced reporter activity driven by the Arg1 3′UTR (Fold change: 0.68 ± 0.08), supporting direct targeting at the predicted sites. Together, these data show that HSV-1 v-miRNAs can directly downregulate Arg-1, IL-10 and IL-2RA via discrete 3′UTR binding sites, supporting a novel post-transcriptional mechanism by which HSV-1 may dampen host immunoregulatory programs during corneal infection.

## 4. Discussion

HSV-1-encoded miRNAs have emerged as important regulators of virus-host interactions, fine-tuning the expression of both viral and host genes (Jurak et al., 2010; Umbach et al., 2009). These v-miRs are abundantly expressed during HSV-1 infection and latency and are transcribed from genomic regions closely linked to the latency-associated transcript, allowing them to modulate local immune responses in infected tissues such as the cornea and trigeminal ganglion (Jurak et al., 2010; Umbach et al., 2009). We previously showed that topical inhibition of HSV-1 miRNAs prevents disease progression by reducing viral replication. In this study, we investigated how the inhibition of viral miRNA (v-miR) affects corneal immune homeostasis. We demonstrated that blocking v-miRs dampens the early inflammatory response by decreasing the recruitment and accumulation of proinflammatory immune cells in the cornea during infection. Consistent with v-miRs directly repressing host transcripts post-transcriptionally, *in vivo* v-miR inhibition results in coordinated upregulation of genes that regulate both myeloid and lymphoid immune responses.

Across the three viral miRNA inhibitors examined (miR-H1–5p, miR-H3–3p, and miR-H6–3p), we observed distinct gene expression changes, indicating that each v-miRNA regulates a different set of host transcripts during HSV-1 corneal infection. The five genes shared across all three inhibitor treatments (Arg1, IL-10, IL-2RA, Adgre1, and CD207) likely represent a common host response pathway modulated by inhibition of these HSV-1 v-miRNAs. Thus, the common gene signature may represent a baseline immunomodulatory effect of v-miRNA inhibition, whereas the greater efficacy observed with specific inhibitors (e.g., miR-H6–3p) is likely driven by broader or more potent regulation of additional inflammatory and antiviral pathways. Notably, miR-H6–3p inhibition exhibited the most pronounced shift in the overall transcriptomic profile, with a clearer transition from proinflammatory toward anti-inflammatory gene signatures compared with the other v-miRs. This data validates our recently published report, which shows that v-miR inhibition not only reduces HSV-1 virion release in the eye washes but also dampens the infiltration of pro-inflammatory cells, including CD4+IFNγ+ T cells and CD4+IL-17+ T cells, in the draining cervical lymph nodes (Patil et al., 2025). The early influx of innate immune cells, particularly macrophages and neutrophils, is critical for limiting viral spread during the initial phase of infection (Marrack and Kappler, 1987, Simmons, 1989). Nash et al. further reported that CD8^+^ T cells contribute to viral control and can be detected in draining lymph nodes of recovering herpetic lesions by day 4 (Nash et al., 1980). We observed a marked reduction in the corneal expression of myeloid cell- and CD8^+^ T cell-specific transcripts in animals treated with miR-H1–5p, miR-H3–3p, and miR-H6–3p inhibitors, suggesting their activity during early HSV-1 infection and thereby reducing viral replication and the production of infectious particles. This reduction would be expected to dampen chemokine signaling, ultimately reducing the recruitment of antiviral T cell responses.

In this study, the HSV-1 miRNAs we examined are derived from the latency-associated transcript (LAT) locus, highlighting LAT as a central source of viral regulatory small RNAs during infection. Importantly, Jones et al. reported that the LAT region produces two additional LAT-encoded small noncoding RNAs, sncRNA1 (62 nt) and sncRNA2 (32 nt) (Peng et al., 2008). Unlike canonical miRNAs, which are typically generated from hairpin precursors through Drosha and Dicer processing and then loaded into Argonaute-containing RISC to direct seed-dependent silencing of target RNAs, these LAT-derived sncRNAs are not classified as miRNAs. However, similar to miRNAs, these virus-encoded small RNAs are capable of reshaping host and viral gene-expression programs, thereby influencing immune responses and pathogenesis. These sncRNAs were demonstrated to shape host-virus interactions by regulating virus persistence and immune responses. For instance, Shen et al. demonstrated that LAT-encoded sncRNA1 and sncRNA2 are expressed during latency and functionally regulate HSV-1 infection by cooperatively inhibiting apoptosis and suppressing productive infection, likely by inhibiting ICP4 levels, consistent with a role in regulating latency–reactivation cycle (Shen et al., 2009). In addition to regulating viral activity, LAT sncRNAs modulate host immune responses by engaging the innate RNA sensor RIG-I to activate IFN-β and NF-κB–dependent transcription (with cell-type specificity for sncRNA2) and, together with RIG-I, suppress apoptosis during HSV-1 infection (da Silva and Jones, 2013). Ghiasi et al. showed that the deletion of the LAT-encoded sncRNA1 did not affect HSV-1 replication or alter latency or reactivation, but it reduced key viral transcripts *in vitro* and lowered LAT expression in trigeminal ganglia during acute infection (Tormanen et al., 2022). Functionally, loss of sncRNA1 increased susceptibility to ocular infection and dampened host innate immune responses, including reduced IFN-β/IFN-γ expression and altered JAK-STAT pathway activation, supporting a protective role for sncRNA1 during primary ocular HSV-1 infection. Together, these observations emphasize that HSV-1 leverages multiple classes of LAT-derived small RNAs, including miRNA and sncRNAs expanding the repertoire of virus-encoded regulatory RNAs that can modulate infection outcomes.

Various studies have shown that TLR9 plays a crucial role in sensing viral double-stranded DNA and triggering antiviral immune responses that promote viral clearance (Lehmann et al., 2012; McGurran et al., 2024; Zou et al., 2022). In our experiments, overexpression of HSV-1 v-miRs in murine macrophages increased phosphorylation of IRF7 and NF-κB compared with a control mimic. Consistent with this, our recent work demonstrated that topical delivery of HSV-1 v-miR inhibitors significantly reduces viral replication in the eye. Together, these results suggest that blocking v-miR activity may dampen TLR9-driven signaling through the IRF7/NF-κB axis, which could help explain the reduced inflammation observed around the eye. In line with this (Daubeuf et al., 2009), reported that TLR9-inhibitory oligonucleotides suppress immediate-early herpes gene expression, consistent with a role for TLR9 signaling in promoting early viral programs. In addition (Li et al., 2024), highlighted the dual role of TLR9 during influenza infection: TLR9 activity appears necessary to initiate an early inflammatory response that supports viral clearance, yet sustained TLR9 activation was also detected in plasma from hospitalized patients and was associated with greater disease severity. Therefore, *in vivo* suppression of v-miRs may attenuate TLR9-driven pro-inflammatory cytokine production, thereby limiting tissue damage caused by excessive inflammation (Miles et al., 2025).

Expression of immunoregulatory and pro-resolving myeloid markers, including *Il-10*, *Il-*2RA (CD25), and arginase (Arg*1*), which are commonly associated with regulatory or resolution-phase macrophage programs, was upregulated in v-miR inhibitor-treated animals. This suggests that relieving miR-H3, miR-H6, or miR-H27–mediated repression amplifies antiviral responses and may restore the corneal immune landscape by enhancing antiviral defense and concurrently upregulating pathways that help limit collateral tissue injury. Consistent with this interpretation, we demonstrated that corneal delivery of v-miR inhibitors induces transcriptional components of the inflammation-resolution machinery, which is required for tissue repair and restoration of homeostasis (Rodríguez-Morales and Franklin, 2023). Specifically, we detected increased expression of resolution-associated lipid mediator pathway genes, including the biosynthetic enzymes *15-lox* and *alox-5*, as well as receptors linked to pro-resolving lipid mediator signaling (SPM-responsive pathways) in corneas treated with miR-H1–5p, miR-H3–3p, and miR-H6–3p inhibitors. The coordinated upregulation of these enzymes and receptors suggests that the v-miRs examined here are potent regulators of resolution-associated programs and that HSV-1 may exploit these miRNAs to perturb activation of resolution pathways, thereby prolonging inflammation and impairing disease resolution.

By directly repressing immune-relevant host transcripts, HSV-1 v-miRNAs can reshape the local antiviral environment in the cornea. V-miR-mediated post-transcriptional regulation can impair myeloid cell activation and antigen presentation, reduce effective T-cell priming, and ultimately limit recruitment of antiviral effector populations into infected tissue. This multilayered control provides a plausible mechanism by which HSV-1 promotes immune evasion and persistence within the ocular microenvironment. Interestingly, our data identify a convergent immunoregulatory signature that emerges when v-miRNA activity is blocked *in vivo*. Specifically, we observed that treatment with miR-H1–5p, miR-H3–3p, and miR-H6–3p inhibitors increased expression of a shared set of anti-inflammatory/pro-resolution genes, including *Il-10*, *Il2RA* and *Arg-1* (and additional immunoregulatory markers). While the full set of experimentally validated host targets for v-miRs, including miR-H1–5p, miR-H3–3p, and miR-H6–3p, remains incomplete, prior work supports the broader concept that herpesvirus miRNAs preferentially target pathways involved in innate immune sensing, cytokine and chemokine signaling, and antigen presentation to dampen antiviral immunity (Skalsky and Cullen, 2010). In this study, we identified and experimentally confirmed three novel functional targets *Il-10*, *Il-*2RA and Arg*1* by HSV-1 miR-H1–5p, miR-H3–3p and miR-H6–3p using dual-luciferase reporter assays. These results reveal a previously undescribed mechanism through which HSV-1 can restrain immunoregulatory pathways that would otherwise promote controlled inflammation and tissue recovery during infection. Restoring IL-10 in the cornea is likely beneficial because IL-10 restrains excessive cytokine and chemokine production by resident and infiltrating immune cells, thereby limiting immunopathology and reducing the risk or severity of HSV-induced stromal keratitis (Deshpande et al., 2001; Suvas et al., 2004). Supporting this concept, prior studies have tested corneal IL-10 gene delivery as a strategy to locally modulate ocular inflammation and track the duration of IL-10 expression (Zhou and Dean, 2007). Similarly, Arg-1 is a hallmark of regulatory/resolution-skewed myeloid programs and can suppress T-cell effector function by depleting arginine and downstream effects on nitric oxide–linked pathways (Karadima et al., 2025). Although this raises the possibility that strong immuno-regulation could impair viral clearance, our prior work has shown that topical inhibition of HSV-1 miRNAs (including miR-H1–5p, miR-H3–3p, and miR-H6–3p) does not increase viral replication but instead reduces viral burden (Patil et al., 2025). Additionally, combined inhibition of multiple HSV-1 v-miRNAs could plausibly produce additive (or synergistic) effects because individual v-miRNAs may converge on overlapping innate immune pathways (e.g., TLR-linked NF-κB and IRF signaling) while also regulating distinct nodes within the same network. In this scenario, blocking a single v-miRNA may only partially relieve pathway dysregulation, whereas combined inhibition could yield a larger net reduction in immune activation and downstream inflammatory pathology. Taken together, these findings suggest that inhibiting HSV-1 v-miRNAs may therapeutically rebalance corneal immunity, preserving antiviral control while restoring IL-10/Arg-1–linked programs that limit tissue-damaging inflammation, promote tissue repair, and restore corneal homeostasis (Recchiuti and Serhan, 2012, Serhan, 2014; Serhan et al., 2015).

In conclusion, our study demonstrates that *in vivo* inhibition of miR-H1–5p, miR-H3–3p, and miR-H6–3p restores corneal immune homeostasis in ocular HSV-1 infection. These viral miRNAs directly target a network of immune-regulatory transcripts in myeloid and lymphoid cells, inducing the expression of various pro-resolution pathway genes. Therefore, therapeutic inhibition of v-miRs reprograms corneal immunity toward robust yet regulated antiviral responses. Future studies will directly test combination regimens to determine whether multi-miRNA inhibition provides benefit beyond the most active single inhibitor.

## Supplementary Material

1

## Data Availability

Data will be made available on request.

## Appendix A. Supplementary data

Supplementary data to this article can be found online at https://doi.org/10.1016/j.exer.2026.110903.
