# Supplementary material for: Viral microRNA inhibition enhances antiviral immunity by modulating corneal inflammatory and resolution pathways in HSV-1 induced keratitis
Source: Exp Eye Res. Author manuscript; Available in PMC 2026 Jul 14. (PMC13367420; doi:10.1016/j.exer.2026.110903)
Supplement: 1 [file NIHMS2191105-supplement-1.docx]

**Viral microRNA Inhibition Enhances Antiviral Immunity by Modulating Corneal Inflammatory and Resolution Pathways in HSV-1 Induced Keratitis**

*Chandrashekhar D. Patil*^#1^, *Raza Ali Naqvi^#^*^2^, *Araceli Valverde*^2^, *Hemant Borase*^1^, *Afsar R. Naqvi*^2,3^, *Deepak Shukla******^1,3^

*^1^Department of Ophthalmology and Visual Sciences, College of Medicine, University of Illinois Chicago, Chicago, Illinois, 60612, USA*

^2^*Department of Periodontics, College of Dentistry, University of Illinois Chicago, Chicago, Illinois, 60612, USA*

^3^*Department of Microbiology and Immunology, College of Medicine, University of Illinois Chicago, Chicago, Illinois, 60612, USA*

***Corresponding Authors:**

Dr. Deepak Shukla

Marion H. Schenk Esq. Professor in Ophthalmology for Research of the Aging Eye

Professor of Ophthalmology, Microbiology and Immunology

Vice Chair for Research; Director, Ocular Virology Laboratory

University of Illinois Chicago

1855 W. Taylor Street, MC 648

Chicago, IL 60612

Phone: 312-355-0908

Fax: 312-996-7773

E-mail address: dshukla@uic.edu

#CDP and RAN contributed equally and are co-first authors.

| **Gene name** | **Forward Primer (5’ to 3’)** | **Reverse Primer (5’ to 3’)** |
| --- | --- | --- |
| **ARG1** | **GCACTCGAGCCCCTATTGACTACCTTAACCCA** | **ATGCGGCCGCTGTGGTAGCTTTGTCTCCTGA** |
| **IL-2RA** | **GCACTCGAGACCAAAAGAACAAGAATTTCTTG** | **ATGCGGCCGCTGTTAACAGCAGAGTGATGACATC** |
| **IL-10** | **GCACTCGAGGACATCAGGGTGGCGACTCTATAG** | **ATGCGGCCGCAATCTGCTATGAAGACAGAC** |

**Supplementary Table 1. Primer sequence for cloning 3’UTRs of target genes.** Underlined sequences showing the restriction sites for Xho1 and Not1.

| **Gene** | **miR-H1-3p**  **(Average Fold change)** | **P value*** |
| --- | --- | --- |
| **Upregulated** | | |
| **Agr1** | 1. 7850908 | 0.044170 |
| **IL-10** | 2.09383737 | 0.020130 |
| **Ccl5** | 1.56206825 | 0.005144 |
| **Ccr2** | 1.61236111 | 0.001696 |
| **Cd80** | 1.05500952 | 0.049201 |
| **IL-2RA** | 1.45893217 | 0.017345 |
| **Csf2** | 1.04186475 | 0.096476 |
| **Fcgrt** | 1.41812352 | 0.000668 |
| **Cd207** | 1.38105625 | 0.098678 |
| **Csf1r** | 1.73615825 | 0.075541 |
| **Csf2** | 1.04186475 | 0.096476 |
| **Fas** | 2.0271795 | 0.098941 |
| **Fcgrt** | 1.4181235 | 0.000668 |
| **Downregulated** | | |
| **Adgre1** | 0.41355775 | 0.007987 |
| **Cd4** | 0.95275675 | 0.012648 |
| **Tlr3** | 0.960176 | 0.007367 |
| **Cd44** | 0.96883775 | 0.741733 |
| **Il21r** | 0.4380405 | 0.054587 |
| **Irf7** | 0.0835195 | 0.000416 |

**Supplementary Table 2. Differential gene expression in mice cornea delivered with inhibitors of HSV-1 derived miR-H1-5p.** *P values for each gene were calculated from multiple t-tests. Cutoff values for upregulation ≥ 1.25 fold and downregulation ≤ 0.75 fold compared to control*

| \| **Gene** \| **miR-H3-3p In**  **(Average Fold change)** \| **P value** \| \| --- \| --- \| --- \| \| **Upregulated** \| \| \| \| **Arg1** \| 1.5844025 \| 0.0080246 \| \| **Ccl19** \| 2.74564775 \| 0.007608 \| \| **Ccr5** \| 1.59054225 \| 0.000431 \| \| **Cd163** \| 1.9967195 \| 0.135623 \| \| **Cd1d1** \| 1.49938725 \| 0.150281 \| \| **Cd3g** \| 1.916436 \| 0.004536 \| \| **Cd4** \| 1.6591646 \| 0.094921 \| \| **IL-2R** \| 2.450690 \| 0.043597 \| \| **Cd8a** \| 2.2363305 \| 0.005294 \| \| **Clec4a** \| 1.627432 \| 0.000322 \| \| **Fcgr3b** \| 1.71422075 \| 0.001067 \| \| **Il15** \| 1.55592825 \| 0.011923 \| \| **Il17Ra** \| 1.462219333 \| 0.04833 \| \| **Il3ra** \| 1.459143333 \| 0.150517 \| \| **Itgae** \| 1.6993785 \| 0.001896 \| \| **Itgax** \| 2.337565 \| 0.000113 \| \| **Ly6g** \| 1.257191 \| 0.802421 \| \| **Mrc1** \| 2.03369525 \| 0.000332 \| \| **Nos2** \| 2.67674425 \| 0.0129518 \| \| **IL-10** \| 3.4534 \| 0.014561 \| \| **Downregulated** \| \| \| \| **Adgre1** \| 0.492277 \| 0.135739 \| \| **Cd207** \| 0.39665175 \| 0.004361 \| \| **Cd209a** \| 0.6346805 \| 0.001912 \| \| **CD226** \| 0.870698333 \| 0.173907 \| \| **CD24a** \| 0.65623725 \| 0.026289 \| \| **Cd40lg** \| 0.79484025 \| 0.099242 \| \| **Cd44** \| 0.726708667 \| 0.013291 \| \| **Cdc42** \| 0.828513 \| 0.001119 \| \| **Cx3cr1** \| 0.6020455 \| 0.004033 \| \| **Fas** \| 0.77449 \| 0.11602 \| \| **Ifnar1** \| 0.684667 \| 0.028514 \| \| **Ldha** \| 0.82877175 \| 0.166134 \| \| **Nono** \| 0.890626333 \| 0.524692 \| |  |  |  |
| --- | --- | --- | --- | --- | --- | --- | --- | --- | --- | --- | --- | --- | --- | --- | --- | --- | --- | --- | --- | --- | --- | --- | --- | --- | --- | --- | --- | --- | --- | --- | --- | --- | --- | --- | --- | --- | --- | --- | --- | --- | --- | --- | --- | --- | --- | --- | --- | --- | --- | --- | --- | --- | --- | --- | --- | --- | --- | --- | --- | --- | --- | --- | --- | --- | --- | --- | --- | --- | --- | --- | --- | --- | --- | --- | --- | --- | --- | --- | --- | --- | --- | --- | --- | --- | --- | --- | --- | --- | --- | --- | --- | --- | --- | --- | --- | --- | --- | --- | --- | --- | --- | --- | --- | --- | --- | --- | --- | --- | --- | --- | --- |
|  |  |  |  |
|  |  |  |  |
| **Supplementary Table 2 : Differential gene expression in mice cornea delivered with inhibitors of HSV-1 derived miR-H3-3p.** *P values for each gene were calculated from multiple t-tests. Cutoff values for upregulation ≥ 1.25-fold and downregulation ≤ 0.75 fold compared to control* |  |  |  |
|  |  |  |  |
|  |  |  |  |
|  |  |  |  |
|  |  |  |  |
|  |  |  |  |
|  |  |  |  |
| \| **Gene** \| **miR-H6-3p (Average Fold change)** \| **P value** \| \| --- \| --- \| --- \| \| **Upregulated** \| \| \| \| **Arg1** \| 204.8799325 \| 0.0040029 \| \| **Arntl** \| 1.489590667 \| 0.172071 \| \| **Bst2** \| 3.629595 \| 0.038951 \| \| **Ccl19** \| 2.98795975 \| 0.144164 \| \| **Ccl5** \| 2.57285225 \| 0.000885 \| \| **Ccr1** \| 3.25002875 \| 0.012806 \| \| **Ccr2** \| 2.14345425 \| 0.047376 \| \| **Ccr5** \| 3.08608725 \| 0.010114 \| \| **Cd163** \| 3.41925875 \| 0.002782 \| \| **CD177** \| 2.9284765 \| 0.000157 \| \| **Cd19** \| 2.61874775 \| 0.015338 \| \| **Cd1d1** \| 5.7737955 \| 0.007547 \| \| **Cd207** \| 4.90903475 \| 0.039811 \| \| **Cd209a** \| 4.544155 \| 0.001299 \| \| **CD22** \| 2.17750675 \| 0.101805 \| \| **CD226** \| 2.9870945 \| 0.000546 \| \| **Cd3g** \| 1.45604175 \| 0.04516 \| \| **Cd40** \| 2.335824 \| 0.119879 \| \| **Cd40lg** \| 4.72806775 \| 0.056543 \| \| **Cd44** \| 1.97515175 \| 0.001216 \| \| **CD69** \| 3.4451175 \| 0.000127 \| \| **Cd74** \| 1.8376505 \| 0.024719 \| \| **Cd86** \| 2.1691675 \| 0.019427 \| \| **Cd8a** \| 2.28693625 \| 0.003445 \| \| **Cx3cr1** \| 2.62841625 \| 0.000303 \| \| **Cxcr1** \| 3.57419125 \| 0.00778 \| \| **Fas** \| 4.290889 \| 0.007885 \| \| **Fcgrt** \| 1.48735625 \| 0.094632 \| \| **H2-Dma** \| 2.96244825 \| 0.004529 \| \| **Icam1** \| 2.45876975 \| 0.018145 \| \| **Icam2** \| 3.009599 \| 0.000133 \| \| **IL10** \| 1.81605775 \| 0.908098 \| \| **Ifnar1** \| 1.87767175 \| 0.008341 \| \| **Il17Ra** \| 1.48322625 \| 0.002289 \| \| **Il3ra** \| 2.4419485 \| 0.021788 \| \| **Irf7** \| 2.1551605 \| 0.019608 \| \| **Isg15** \| 2.19404525 \| 0.070636 \| \| **Itgae** \| 1.58381275 \| 0.006225 \| \| **Itgam** \| 2.00798575 \| 0.018133 \| \| **Itgax** \| 1.37946825 \| 0.040396 \| \| **Kir2dl1** \| 3.3223105 \| 0.00631 \| \| **Kir2dl4** \| 4.37829875 \| 0.018721 \| \| **Ly6g** \| 2.51723125 \| 0.015249 \| \| **Nos2** \| 3.03942525 \| 0.017128 \| \| **Ldha** \| 1.56218275 \| 0.068738 \| \| **Tap2** \| 1.9840675 \| 0.0191 \| \| **Nos** \| 2.4304081 \| 0.0490754 \| \| **Tapbp** \| 1.67012825 \| 0.040587 \| \| **Mrc1** \| 2.81901075 \| 0.001646 \| \| **Mx1** \| 4.0755415 \| 0.012529 \| \| **Tgfb1** \| 1.48651775 \| 0.11519 \| \| **Csf2** \| 4.197248 \| 0.019624 \| \| **Downregulated** \| \| \| \| **CD24a** \| 0.79121075 \| 0.052714 \| \| **Klrc2** \| 0.62065975 \| 0.013121 \| \| **Nfkb1** \| 0.6935655 \| 0.012181 \| \| **Rorc** \| 0.937497 \| 0.009092 \| \| **Adgre1** \| 0.38742025 \| 0.007132 \| |  |  |  |

**Supplementary Table 3 : Differential gene expression in mice cornea delivered with inhibitors of HSV-1 derived miR-H6-3p***. P values for each gene were calculated from multiple t-tests. Cutoff values for upregulation ≥ 1.25 fold and downregulation ≤ 0.75 fold compared to control*

**
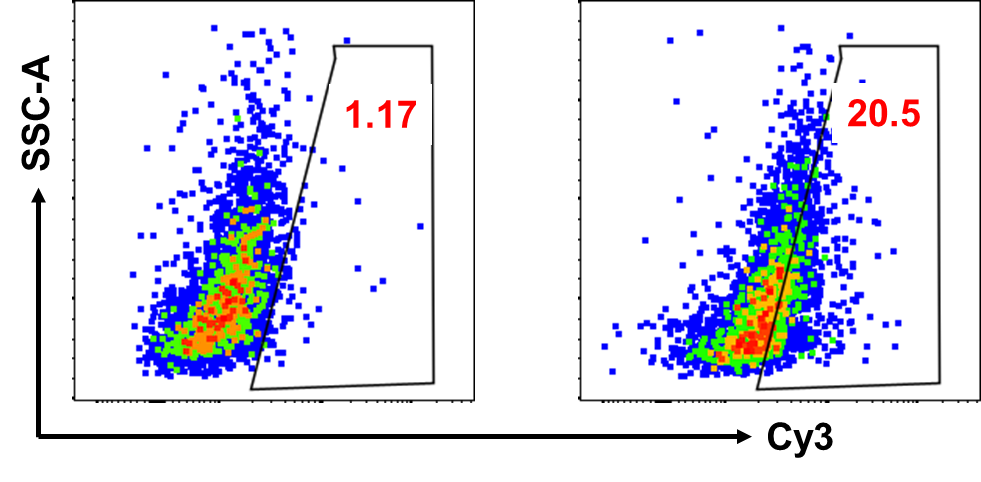
**

**Supplementary Figure 1: *In vivo* uptake of a Cy3-labeled vmiR inhibitor.** To assess tissue uptake and penetration, we performed *In Vivo* uptake experiments using a Cy3-labeled vmiR inhibitor after topical administration to mouse corneas. Corneal tissues were harvested, dissociated into single-cell suspensions, and analyzed by flow cytometry.
